# The paramedics’ pledge: a short commentary on its inception and development

**DOI:** 10.29045/14784726.2023.12.8.3.52

**Published:** 2023-12-01

**Authors:** Lawrence Hill, Georgette Eaton

**Affiliations:** University of East Anglia ORCID iD https://orcid.org/0000-0002-9147-0934; University of Oxford; London Ambulance Service NHS Trust ORCID iD: http://orcid.org/0000-0001-9421-2845

**Keywords:** paramedics’ pledge, philosophy of practice, professional identity

## Abstract

This article discusses the creation and evolution of a unifying pledge designed to encapsulate the identity of paramedics and convey the essence of belonging to this professional group.

## Background

The paramedic profession is developing at pace, and becoming increasingly professionally diverse. This is fantastic for us as individual paramedics, and for our profession, with so many opportunities opening up as the scope of role (and practice) evolves. Yet, as this happens, expectations placed on paramedics are changing, particularly in healthcare. Political forces are influencing the placement exposure of students, and market forces are dictating where paramedics find themselves working clinically. Despite this stretching of the conventional notion of ‘the paramedic’, there seems to be an essence that unites us beyond the title of ‘paramedic’.

And yet, this essence does not appear to have been captured anywhere. What it means to *be* a paramedic is not something that is articulated in existing definitions of the profession, defined in scope-of-practice policies or generated by regulatory standards, even though there are features of becoming, being, knowing and doing for paramedics that appear to be universal, and that resonate internationally.

We wanted to create something that captures the essence of ‘who we are’ as paramedics, and that speaks to us as individuals and as occupants of a unique and increasingly diverse societal role/group.

## Transcendental paramedics

We looked at other organisations, groups of people and professions that have stood the test of time and surfed the waves of change. Medicine has an oath, Girlguiding and the Scout Association have a promise and firefighters have a prayer – all of which serve to bring together autonomous and diverse members into one body and to simultaneously define their place in society. These oaths, promises and prayers describe their essence, and give a sense of what it means to ‘belong’ to that group.

We think that what paramedics do is no less important and no less noble than other institutions that have defined their place in our global society. So, we wrote a pledge in an attempt to philosophically elevate the paramedic profession from a pre-dominantly epistemological and vocational domain concerned with ‘knowing’ and ‘doing’, to a more ontological and existential domain concerned with the reality of ‘becoming’ and ‘being’. In doing so, we hope to provide a way for paramedics of increasing professional diversity and specialisation to retain an aspect of identity that they can put their finger on and say, ‘Yes, that is me, I am a paramedic’.

### The hatching and nurture of the paramedics’ pledge

The paramedics’ pledge was drafted as a product of the thinking and evidence relating to Lawrence Hill’s doctoral research into paramedic professional identity. Through the College of Paramedics (UK), Georgette Eaton came on board with the early development. The College of Paramedics subsequently nurtured the paramedics’ pledge and worked with us to ensure it was exposed to a wide array of critical voices from within the international paramedic community. And this is important – the paramedics’ pledge was not written *just* by us. During the consultation with the College of Paramedics, we spoke with paramedics from across the United Kingdom and across the world, paramedics working in diverse settings, paramedics at the beginnings and the endings of their careers. We heard their voices; we shared their stories. Through this, the paramedics’ pledge contains the opinions and perspectives of so many paramedics from across the globe – all of whom we would like to thank for their thoughts, their words of challenge and their wisdoms. Lawrence and Georgette are the curators, rather than creators, of the paramedics’ pledge. This belongs to all of us.

### The fledging of the pledge

In May 2023, the paramedics’ pledge was presented at the College of Paramedics National Conference and at the International Roundtable Committee on Community Paramedicine, in Nottingham, England. On 8 July 2023, the paramedics’ pledge was launched at International Paramedics’ Day 2023. This article means that paramedics can now reference the paramedics’ pledge as part of the academic literature within our profession.

For some, we know the idea of pledge has value – and we know that for others it does not. Each of our professional identities is unique to us and for some the paramedics’ pledge will seem unnecessary – and we respect that. To others, we know it provides a statement that speaks to the person at the centre of their paramedic identity.

For those with whom the paramedics’ pledge resonates – wherever you are in the world – you know there are others out there who share with you a sense of purpose and a sense of pride in this professional title. You stand in solidarity with paramedics who share with you what it means to know what you know, to do what you do and be what you are.

## The paramedics’ pledge

I swear to fulfil, to the best of my ability, this pledge – that binds together my curiosity, compassion, and humility, with my duty as a paramedic

I recognise that I do not stand alone but alongside all those who share my professional purpose. Together, we walk proudly in the footsteps of those whose endeavours have developed our profession

And yet, we remain inquisitive, carving our own path in pursuit of progress towards the betterment of the communities we serve

Though we are adaptable and operate with autonomy, we do not work in isolation – conscious that our very name means that we work alongside others

Our complex and austere experiences give us insight. Using this wisdom, we do everything reasonable, within the boundaries of what is ethical and lawful, to benefit those in our care

We tread with care in all matters of life, including death, creating enough proximity to afford empathy yet preserving enough distance to maintain a professional boundary

We take pride in the privilege of our position. We seek to protect the rights, interests, and dignity of our fellow humans because it is our role to advocate for all people – regardless of how their values may differ from, or echo, our own

Although we are altruistic, we also accept our duty to be compassionate to ourselves because we are mindful that the virtues that we strive to uphold may, at times, weigh heavily upon us

And if we are true to ourselves and to this pledge – then let us be known as a profession who act with integrity and equanimity in the face of challenge and complexity

Our ethos emerges from our experiences, is moulded by our curiosity, moderated by our humility, and animated by our compassion –

We are paramedics.

## Using the paramedics’ pledge

Please use the paramedics’ pledge! The most important part of it is the message that we are a profession united by our experiences and our values. We have a couple of suggestions of how you might like to use the paramedics’ pledge, but imagine you will have other ways you can too. Our thoughts are:

As a student:

- Something to aspire to.
- Something to use as a grounding statement to guide your efforts and keep your momentum.

As an individual:

- Something you can read if you need to be reminded that what you do is profound and important.

As an educator:

- Something to talk to your students about as you assist them to develop their professional identity.
- Something to be read at graduations, badge ceremonies, passing-out parades, celebrations of achievement.
- Something to present to paramedics for outstanding service.

As a company or service:

- Something to hang in social areas.
- Something to affiliate your organisation with to show your commitment to paramedics.

As a regulator, professional body or governing board:

- Something that could be used to bind individual professional values with an organisational philosophy.

However you use it, we would love to hear about it!

The paramedics’ pledge is covered under Attribution-NonCommercial-NoDerivatives 4.0 International. The academic reference for the paramedics’ pledge is:

Hill, L., & Eaton, G. (2023). The paramedics’ pledge: A short commentary on its inception and development. *British Paramedic Journal, 8*(3), 52–54.

## Author contributions

The paramedics’ pledge was drafted as a product of the thinking and evidence relating to Lawrence Hill’s doctoral research into paramedic professional identity. Through the College of Paramedics (UK), Georgette Eaton came on board with the early development. Both authors contributed equally to the conception and development of this article. LH acts as the guarantor for this article.

## Conflict of interest

None declared.

## Funding

None.

